# Unmet expectations: social inclusion and the interaction between social anxiety and ambiguous or positive feedback

**DOI:** 10.3389/fpsyg.2023.1271773

**Published:** 2023-12-05

**Authors:** Rémi Thériault, Flavie Dion-Cliche, Stéphane Dandeneau

**Affiliations:** Department of Psychology, Université du Québec à Montréal, Montréal, QC, Canada

**Keywords:** social inclusion, social participation, fundamental needs, fear of negative evaluation, social anxiety, Uberball, Cyberball

## Abstract

**Introduction:**

This study explores the impact of preferential inclusion on fulfilling basic needs following ambiguous or positive social feedback, considering the moderating effect of social anxiety.

**Methods:**

Participants (*N* = 438) received either positive or ambiguous social feedback and engaged in a social participation or preferential social inclusion task. They completed measures of the fulfillment of their fundamental needs, social anxiety, and other personality traits.

**Results:**

The results indicate that preferential social inclusion (Uberball condition) enhances the fulfillment of fundamental needs compared to social participation (Cyberball inclusion condition). Furthermore, receiving positive social feedback considerably strengthens the negative relationship between social anxiety and fundamental need fulfillment when followed by ordinary social participation relative to preferential social inclusion presumably because these individuals react more strongly to unmet expectations of extreme social acceptance.

**Discussion:**

This research suggests that individuals with high social anxiety may not experience the usual benefits of social participation unless they experience extreme social inclusion.

## Introduction

Interpersonal acceptance and rejection have powerful psychological consequences. Social acceptance is vital to wellbeing, whereas social exclusion causes negative emotions and hinders psychological health (Leary and Baumeister, 2000; Williams et al., 2000; Williams, 2007; Leary, 2010; Williams and Nida, 2011; Hales and Williams, 2021). Explicit cues that others dislike or reject us are also among the most powerful contributors to feelings of low self-esteem (Leary and Baumeister, 2000). Being abandoned, romantically rejected, or excluded from social groups are highly distressing events, usually followed by self-esteem drops (Leary and Baumeister, 2000). Moreover, social rejection thwarts one’s fundamental needs of belonging, meaningful existence, self-esteem, control, and certainty, leading to negative emotional, cognitive, behavioral, and neural consequences (Williams et al., 2000; Eisenberger et al., 2003; Williams, 2007; Williams and Nida, 2011; Hales and Williams, 2021).

To experimentally manipulate or induce feelings of ostracism, Williams and colleagues developed the Cyberball paradigm, where participants play a ball-tossing game on the computer in which they experience either social exclusion (other players stop throwing the ball to the participant) or social “inclusion” (other players throw the ball to the participant and other players equally; Williams et al., 2000; Williams and Jarvis, 2006). The main purpose of the Cyberball paradigm was to investigate to effect of ostracism. Despite the wealth of knowledge on the effects and consequences of social rejection, little empirical research has investigated the effects and conditions needed to promote and increase feelings of social *acceptance*. Until recently, the Cyberball *inclusion* condition was assumed to have the opposite effects of the Cyberball *exclusion* condition. Being included in the ball-tossing game would increase participants’ fundamental needs of self-esteem, belonging, meaning, control, and certainty (Hales and Williams, 2021). However, researchers showed that this was not the case and that the Cyberball *inclusion* condition is more akin to a control social participation task (Simard and Dandeneau, 2018; Dvir et al., 2019).

Making participants the *specific* target of inclusion while another player is excluded (an inclusion condition called *Uberball*) showed significant *increases* in fundamental need fulfillment (of belongingness, self-esteem, meaningful existence, but not control; Simard and Dandeneau, 2018) relative to a neutral control condition, whereas the standard Cyberball *inclusion* condition did not. In other words, it seems that increasing feelings of acceptance, above and beyond pre-existing levels, requires more than simply “participating or being included” in a game—it requires explicit and overt cues, indicating that we are “chosen” as part of the group. These effects were not explained by the participant’s feelings of sympathy toward the “excluded” participant or mood but rather by the overt social cues that the others chose *them* and not someone else. Furthermore, the positive effects of the preferential inclusion of the Uberball condition were strongest for participants with relatively high levels of social anxiety (and null for participants with low levels of social anxiety), suggesting that unambiguous social cues of social inclusion benefit those who tend to construe their context as a socially hostile environment (Simard and Dandeneau, 2018).

These results highlight two important aspects of social functioning (1) that merely participating in a social task does not seem to boost feelings of group acceptance—one needs clear and overt cues that indicate one’s inclusion to feel an increase in acceptance from baseline, and (2) clear and overt inclusion may counteract the negative interpretation bias shown in socially insecure individuals. One cognitive factor that contributes to social anxiety is the tendency to interpret ambiguous social information negatively. A recent systematic review and meta-analysis indicates that socially anxious individuals hold a negative or threat bias for ambiguous social situations and report catastrophic interpretations of mildly negative social situations (Chen et al., 2020). It also provides evidence for the cognitive theoretical framework that socially anxious individuals interpret ambiguous social information more threateningly than non-anxious individuals (Chen et al., 2020; see also Beard and Amir, 2008).

Ambiguous social information can be perceived as a social threat due to the brain’s propensity to prioritize negative information in social contexts. According to the negativity bias theory, negative information, such as ambiguous social cues, is more salient and impactful on an individual’s emotional and cognitive processes than positive information (Baumeister et al., 2001). Ambiguity is inherent in many social interactions, and individuals must rely on social cues, such as facial expressions, tone of voice, and body language, to navigate and interpret these interactions. For some people, namely, those with highly sensitive and anxious social radars, ambiguity in these social interactions can result in misinterpretation and negative perceptions of social situations (Amir et al., 2005; Yun and Hyun, 2023). Ambiguity can lead to uncertainty, triggering anxiety and stress in individuals, especially those with heightened social vulnerability (Carleton et al., 2006). Furthermore, social ostracism is also frequently experienced as an ambiguous experience that threatens feelings of certainty (Hales and Williams, 2021). It is thus possible that ambiguous social feedback is perceived and experienced as social ostracism.

Research has shown that individuals with high anxiety and social anxiety levels are particularly susceptible to interpreting ambiguous social information as threatening. For example, one study found that individuals with high social anxiety were more likely to interpret neutral faces as threatening, suggesting they have a heightened sensitivity to ambiguous social cues (Hirsch et al., 2006). Moreover, individuals with social anxiety often have negative self-evaluations and a fear of negative evaluation by others. This leads to a heightened sensitivity to ambiguous social cues that may be perceived as social threats (Heinrichs and Hofmann, 2001).

The perception of ambiguous social information as a social threat may also stem from one’s cognitive biases, that is, systematic errors in thinking that can influence perception, judgment, and decision-making (Tversky and Kahneman, 1974). One such cognitive bias is the confirmation bias, where one tends to seek out information that confirms pre-existing beliefs or attitudes and ignore information that contradicts them. In this light, individuals with social anxiety may be more likely to display a confirmation bias when interpreting ambiguous social cues, leading to a greater likelihood of perceiving such cues as threatening (Carleton et al., 2007), possibly influencing their behavior in a self-fulfilling prophecy fashion (Stinson et al., 2009, 2011).

In the current study, we extend previous research by integrating positive and ambiguous social feedback (Anthony et al., 2007; Schröder-Abé et al., 2007; Yang and Girgus, 2018) with the Uberball condition to test whether fortifying participant’s fundamental needs can mitigate the effects of ambiguous social feedback. Social ostracism seems to motivate people to restore their basic needs and make them more sensitive to future social information (Hales and Williams, 2021); therefore, in the current study, participants should be particularly sensitive to the group’s inclusive behavior following ambiguous feedback. We reasoned that if ambiguous feedback strongly affects socially insecure individuals, these individuals would benefit the most from the Uberball condition’s restorative power, consistent with previous research (Simard and Dandeneau, 2018). We thus initially predicted that fostering preferential inclusion (e.g., through the Uberball condition) after receiving ambiguous social feedback would strengthen the fundamental needs of socially anxious participants.

On the other hand, we also recognize that the complicated nature of social anxiety and acceptance makes this prediction rather simplistic, given that social anxiety may change the way individuals interpret social interactions relative to non-anxious individuals. Indeed, previous research did not include a positive or ambiguous feedback manipulation *prior* to experiencing social inclusion; therefore, it is plausible that the effect would not be strictly linear. In particular, as mentioned earlier, the dynamics of confirmation bias and self-fulfilling prophecies in socially anxious individuals may, on the one hand, make them less receptive to future experiences of inclusion—even overinclusion—and under-detect acceptance if they have been primed with potentially ostracizing ambiguous social feedback (e.g., Cameron et al., 2010).

### Present study

This study aimed to replicate and extend previous findings regarding the preferential social inclusion condition of Uberball. Based on previous research (Simard and Dandeneau, 2018), we hypothesized that the Uberball condition (vs. the Cyberball inclusion condition) would lead to higher fulfillment of fundamental needs and perceived relational value. Our central objectives were to test (1) whether experiencing preferential social inclusion (Uberball condition) mitigates the adverse effects of ambiguous social feedback on the fulfillment of fundamental needs and perceived relational value and (2) whether participants’ level of fear of negative evaluation moderates this effect. Specifically, we predicted that those *high* in fear of negative evaluation would benefit more from the Uberball condition than the Cyberball inclusion condition after receiving *ambiguous* feedback. We did not expect such an effect when the feedback is *positive* or for those with a *low* fear of negative evaluation.

## Method

### Participants and design

The sample size was determined before any data analysis. Power analyses with an alpha level of 0.05 and 80% power suggested sample sizes of at least 104 per group (208 total) for *t*-tests (with an expected small-medium Cohen’s *d* effect size of 0.39 based on Simard and Dandeneau, 2018) and at least 395 for moderation analyses (with an expected small *f*^2^ of 0.02 for the three-way interaction). We recruited five hundred participants through Amazon Mechanical Turk to participate in the online study, anticipating the loss of approximately one-third of the data due to incomplete or missing data (Litman et al., 2017). We excluded data from 16 participants due to incomplete or invalid data, 10 for failing the attention check, and 36 for knowing the purpose of the Cyberball paradigm before starting the experiment. This left 438 participants (58.7% women) with a mean age of 39.0 years (*SD* = 12.1 years) for the analyses (61% from the USA, 31% missing location data, and 8% from other countries). No demographics on racial/ethnic identity or language spoken were collected. Sensitivity analyses suggested such a sample size provided sufficient power to detect Cohen’s *d* effects greater than 0.26 (for main effects comparing two collapsed groups of 219 in each condition) and *f*^2^ effects greater than 0.017.

The study consisted of a 2 (Feedback condition: Positive vs. Ambiguous) × 2 (Inclusion Type: Uberball condition vs. Cyberball condition) between-subject design where participants were randomly assigned to one of the four combinations of conditions. We report all tasks and measures below.

### Conditions

#### Feedback conditions

Based on Anthony et al. (2007) methodology, we presented positive or ambiguous feedback relative to participants’ involvement in an upcoming group task. Participants were asked to answer (yes/no) to the following questions: “Do you like heavy metal music? Do you tend to give money to homeless people? Are you a sports person? Do you like going to amusement parks?” ostensibly to provide a brief “profile” to their team members. Participants were asked to wait 1 min while the system compiled responses from their group members, and during this time, they viewed other team members’ “answers” to the same questions.

Participants in the *positive feedback* condition (*n* = 212) were told that other participants responded to their profile questions with the following responses: “This person seems nice. I hope she will join us.” “This person sounds nice. I’m looking forward to working with them,” or “I think she’ll really gel with the group in no time at all.”

Participants in the *ambiguous feedback* condition (*n* = 226) were told that other group members’ responses to their profile were “We seem pretty different, but I’m willing to give it a try.,” “I think we’ll get along well after we really get to know each other.,” or “This person sounds like someone I could grow to like.”

#### Social inclusion conditions

We manipulated participants’ feelings of inclusion with the Cyberball inclusion and the Uberball inclusion conditions. The *Cyberball inclusion* condition (*n* = 232) consisted of the 4-player version of the Cyberball inclusion online ball-tossing game where all participants are given approximately the same percentage of throws throughout the game (33%) (Williams et al., 2000).

The *Uberball Inclusion* condition (*n* = 206) is identical to the Cyberball inclusion condition; however, after approximately five throws, the preprogrammed players to the left and atop the participant *only start sending throws to the participant* (and stop sending throws to the player to the right of the participant). The participant can send throws to whomever they wish (left, atop, or right). This condition clearly and overtly indicates to the participant that *they* are the target of preferential social inclusion as they receive about 90% of the throws (Simard and Dandeneau, 2018).

The Uberball and Cyberball inclusion conditions consisted of 50 throws that lasted approximately 5 min and were programmed using Inquisit Web software (Millisecond Software LLC, 2016).

### Measures

#### Anticipated group acceptance

Anticipation of being accepted by the group was measured on a scale designed from items used in the study by Anthony et al. (2007) as well as from other items created for this study (example item: “How likely is it that the others will like you?”). This 9-point scale ranged from *not at all* to *very much* (*α* = 0.95). This measure was used as a manipulation check, following the ambiguous and positive feedback manipulations.

#### Fundamental needs

The fundamental needs of belonging, self-esteem, meaningful existence, and control were assessed using a 5-point scale ranging from *not at all* (1) to *extremely* (5; Jamieson et al., 2010). A total mean score was computed (*α* = 0.94), where a higher score indicates a higher level for each need, that is, more *fulfilled* needs (example items for *belonging*, “I felt I belonged to a group”; *self-esteem*, “I felt liked and worthy”; *meaningful existence*, “I felt important”; and *control*, “I felt powerful”). The overall score (mean of 4 subscales) and the four individual subscales were used as our primary dependent measures.

#### Perceived relational value

Perceived relational value was assessed using a 7-point scale ranging from *not agree at all* (1) to *very strongly agree* (7; Simard and Dandeneau, 2018). A total mean score was computed (*α* = 0.96; example item: “I felt like others value playing with me”). This measure was used as an additional outcome measure.

#### Fear of negative evaluation

Participants’ fear of negative evaluation was assessed using Carleton et al.’s (2006) 5-point scale ranging from *Not at all characteristic of me* to *Extremely characteristic of me* (Carleton et al., 2006). Higher scores indicate a high fear of negative evaluation (*α* = 0.95; example item: “I am frequently afraid of other people noticing my shortcomings”). As was the case in Simard and Dandeneau (2018), this measure was used in our primary moderation analyses.

#### Other measures

We also took measures of self-esteem (Rosenberg, 1965), rejection sensitivity (Downey and Feldman, 1996), relational security with friends (Stinson et al., 2011), and mood (Kercher, 1992) for exploratory purposes. All measures used in this study (including exploratory measures) are available in Supplemental Materials.^1^ In this study, we report all measures, manipulations, and exclusions.

### Procedure

Participants first read the description of the study and provided informed consent and demographic information. They also completed the brief version of the Fear of Negative Evaluation Scale (Carleton et al., 2006) and other personality measures (e.g., Rosenberg Self-Esteem Scale and Rejection Sensitivity Scale). Participants then read the same experimental vignette asking them to imagine themselves in a first impression context involving three other people (i.e., the other three players in the Cyberball paradigm). As a result, each participant was required to disclose personal information (e.g., hobbies and employment) and was then asked to assess the same information provided by “others” (the other’s information was in fact pre-scripted). Participants were then randomly assigned to one of the four experimental groups where they first received feedback (e.g., positive or ambiguous), completed the measure of anticipation of their social acceptance of the group, and then completed either the Uberball or Cyberball social inclusion conditions. Finally, participants completed measures of fundamental needs, relational value, relational security with friends, and mood and were debriefed and thanked for participating.

### Analyses

As per recommendations, we report item-level missing values by scale and the participant’s maximum number of missing items by scale (Parent, 2013). Fear of negative evaluation had 0.59% missing data points (with no participant with more than three missing items); anticipation: 0.40% missing (max two missing items); relational value: 0.40% missing (max one missing item); and fundamental needs: 0.56% missing (max five missing items). Visual inspection of the missing data revealed no specific patterns. Little’s test confirmed this interpretation by failing to reject the null hypothesis that the missing data were missing completely at random. As per best practices (van Ginkel et al., 2020), we imputed item-level missing values (before calculating the scales’ means) via the *missForest* package (Stekhoven and Bühlmann, 2012; Stekhoven, 2022). To ensure optimal normal distribution of the data, we identified and applied optimal normalizing transformations (one of Box-Cox or Yeo-Johnson) via the *bestNormalize* package (Peterson and Cavanaugh, 2020; Peterson, 2021). We used Welch *t*-tests per recommendations (Delacre et al., 2017). The transformed data satisfactorily met all the univariate and model-based assumptions, and there were no outliers based on three median absolute deviations (Leys et al., 2013; Thériault et al., 2023a). We report raw descriptive statistics (before transformations) of all relevant variables in Table 1.

**Table 1:** Descriptive statistics.

Variable	Mean	SD	IQR	Min	Max	Skewness	Kurtosis	*n*	Missing
Age	39.02	12.08	17.00	19.00	73.00	0.73	−0.19	436	2
Fear of negative evaluation	2.85	1.06	1.58	1.00	5.00	0.13	−0.86	438	0
Anticipation	6.86	1.49	2.03	1.25	9.00	−0.71	0.41	438	0
Needs	3.78	0.75	1.05	1.05	5.00	−0.72	0.42	438	0
Need to belong	3.93	0.84	1.05	1.00	5.00	−0.86	0.51	438	0
Need for self-esteem	3.88	0.85	1.00	1.20	5.00	−0.73	0.30	438	0
Need for meaningful existence	4.08	0.83	1.00	1.00	5.00	−1.16	0.90	438	0
Need for control	3.23	0.84	1.00	1.00	5.00	−0.19	−0.03	438	0
Relational value	5.12	1.52	2.00	1.00	7.00	−0.76	−0.16	438	0

We performed all statistical analyses in R version 4.2.0 (R Core Team, 2022) using the following additional packages: *visdat* (visualizing missing data; Tierney, 2017), *naniar* (Little’s MCAR test; Tierney et al., 2021), *pwr* (power analyses; Champely, 2020), *lmSupport*, *bootES*, and *effectsize* (effect sizes and bootstrapped confidence intervals; Kirby and Gerlanc, 2013; Curtin, 2018; Ben-Shachar et al., 2020, 2022), *interaction* (moderations and figure; Long, 2019), *psych* (internal reliability analyses; Revelle, 2018), *dplyr* (data manipulation; Wickham et al., 2021), *ggplot2* (Wickham, 2016), *rcompanion* (Mangiafico, 2020), *ggsignif* (Ahlmann-Eltze, 2019), *ggrepel* (Slowikowski et al., 2018), and *ggpubr* (Kassambara, 2019) for figures, as well as report (Makowski et al., 2022) and *rempsyc* (Thériault, 2023) for convenience functions. The data and analysis scripts are available on the Open Science Framework at https://osf.io/cmg3z/.

## Results

A manipulation check *t* test revealed a statistically significant effect between the ambiguous and positive feedback groups on anticipation of social acceptance (*M*_Ambiguous_ = 6.57, *M*_Positive_ = 7.18; difference = 0.62, 95% CI [−0.89, −0.34]),^2^ *t*(427.69) = −4.78, *p* < 0.001; (Cohen’s *d* = −0.46, 95% CI [−0.65, −0.27]), suggesting that the feedback manipulation created different levels of anticipated social acceptance.

### Replication analyses

The first analyses consisted of the same analyses reported by Simard and Dandeneau (2018) to see whether (a) participants in the Uberball condition (compared to the Cyberball inclusion condition) showed higher levels of fundamental need fulfillment and perceived relational value and (b) a moderation of fear of negative evaluation between inclusion condition and fundamental needs.

**Figure 1:**
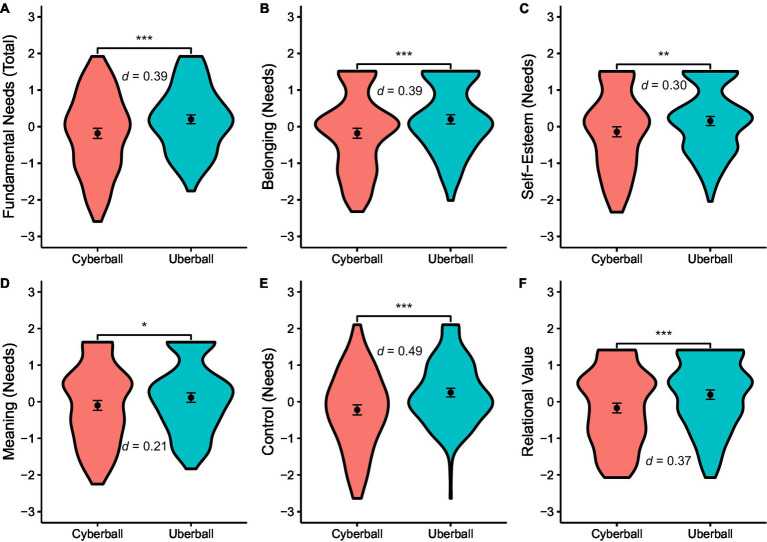
Violin plots of fundamental needs (**Panels A-E**) and relational value (**Panel F**). Violin plots comparing Cyberball inclusion and Uberball on fundamental need fulfillment and perceived relational value. Dots, means; error bars, bootstrapped 95% confidence intervals; width, distribution density (frequency). ***, *p* < 0.001; **, *p* < 0.01. This plot uses the transformed (and standardized) data.

Regarding result (a), the Uberball condition does lead to higher fulfillment of fundamental needs (overall needs and individual needs) and perceived relational value than Cyberball inclusion, as in previous research and with comparable effect sizes (Table 2; Figure 1).

**Table 2:** Results of pairwise comparisons (Cyberball inclusion vs. Uberball) on fundamental needs and relational value.

Dependent variable	Subdimension	*t*	*df*	*p*	*d*	95% CI
Fundamental needs	**Total Needs**	**4.14**	**433.60**	**<0.001**	**0.39**	**[0.20, 0.58]**
	**Belonging**	**4.07**	**435.78**	**<0.001**	**0.39**	**[0.20, 0.58]**
	**Self-Esteem**	**3.16**	**434.95**	**0.002**	**0.30**	**[0.11, 0.49]**
	**Meaning**	**2.28**	**435.92**	**0.023**	**0.22**	**[0.03, 0.40]**
	**Control**	**5.14**	**432.33**	**<0.001**	**0.49**	**[0.30, 0.68]**
Relational value	…	**3.89**	**435.37**	**<0.001**	**0.37**	**[0.18, 0.56]**

*d*, Cohen’s d; CI, confidence interval. The transformed (and standardized) data were used in the analyses reported in this table. Shaded/bolded areas represent statistically significant rows.

Regarding results (b), a critical difference between Simard and Dandeneau (2018) studies and the current study is that participants in the present study underwent a positive/ambiguous feedback manipulation *before* completing the Uberball/Cyberball inclusion conditions. The fear of negative evaluation by condition interaction on fundamental need fulfillment was not significant (*β* = 0.08, *t*(434) = 0.93, *p* = 0.354, *sr*^2^ = 0.00 [0.00, 0.01]).^3^ Visual assessment of the data revealed that all participants, regardless of levels of fear of negative evaluation and feedback group, seemed to have benefited from the Uberball condition relative to the Cyberball inclusion condition. This discrepancy with Simard and Dandeneau (2018) is not totally unexpected, however, given that in our study, participants received social feedback beforehand, which could have changed the effect that the fear of negative evaluation × Uberball condition interaction has on fundamental needs. The next section addresses this point by demonstrating a three-way interaction between fear of negative evaluation, social feedback condition, and inclusion condition.

### Primary analyses

Our main hypotheses tested the two-way “feedback × condition” and the three-way “feedback × condition × fear of negative evaluation” interactions on fundamental needs as the dependent variable. We used general linear and simple linear moderation models to examine these hypotheses.

**Figure 2:**
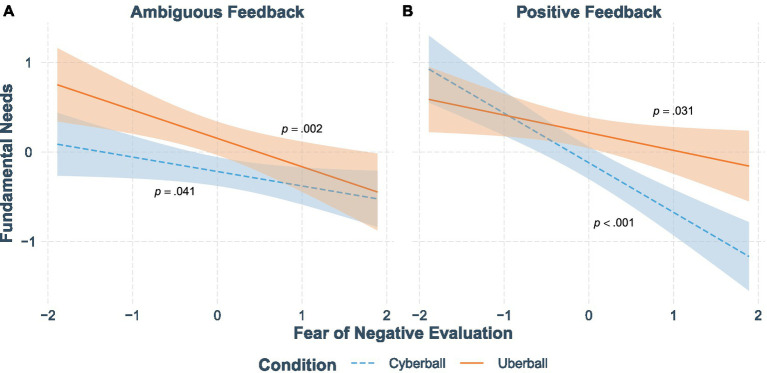
Simple slopes of fundamental needs for participants in the ambiguous (**Panel A**) and positive (**Panel B**) feedback groups in the Cyberball inclusion and Uberball conditions. Error bands represent 95% confidence bands. *p*-values are for the simple slope analyses for the difference between Cyberball inclusion and Uberball (*x*-axis). This plot uses the transformed (and standardized) data.

First, in contrast to our hypotheses, the two-way interaction “feedback × condition” was not significant (Table 3). As predicted, the “feedback × condition × fear of negative evaluation” interaction term significantly predicted fundamental needs (Table 3). However, the nature of the interaction differs from our predictions. To decompose this complex three-way interaction, we tested a two-way interaction for each of the ambiguous feedback and positive feedback conditions separately. As suggested by Figure 2, the two-way interaction between inclusion condition and fear of negative evaluation is significant only in the positive feedback condition (*β* = 0.36, *t*(208) = 2.83, *p* = 0.005, *sr*^2^ = 0.03 [0.00, 0.07]) but not in the ambiguous feedback condition (*β* = −0.16, *t*(222) = −1.20, *p* = 0.233, *sr*^2^ = 0.01 [0.00, 0.02]).

**Table 3:** Results of multiple regression analyses.

Dependent variable	Predictor	*df*	*β*	*t*	*p*	*sr*^2^	95% CI
Fundamental needs	Feedback	430	0.10	0.79	0.430	<0.00	[0.00, 0.01]
	**Condition**	**430**	**0.37**	**2.95**	**0.003**	**0.02**	**[0.00, 0.04]**
	**Fear of negative evaluation**	**430**	**−0.16**	**−2.05**	**0.041**	**0.01**	**[0.00, 0.02]**
	Feedback × Condition	430	−0.03	−0.19	0.847	<0.00	[0.00, 0.00]
	**Feedback** × **Fear of negative evaluation**	**430**	**−0.39**	**−3.29**	**0.001**	**0.02**	**[0.00, 0.05]**
	Condition × Fear of negative evaluation	430	−0.16	−1.21	0.226	<0.00	[0.00, 0.01]
	**Feedback** × **Condition** × **Fear of negative evaluation**	**430**	**0.51**	**2.83**	**0.005**	**0.02**	**[0.00, 0.04]**

*β*, standardized regression coefficient; *sr*^2^, semi-partial correlation squared; feedback, social feedback (ambiguous vs. positive); condition, inclusion condition (Cyberball inclusion vs. Uberball); transformed (and standardized) data were used in the analyses reported here. Shaded/bolded areas represent statistically significant rows.

To further explore the interaction, we conducted simple slope analyses for each feedback condition separately (with +1/−1 SD; Aiken and West, 1991; Hayes, 2018). In the *ambiguous* feedback analyses, there was a significant condition effect (Cyberball inclusion condition vs. Uberball condition) on fundamental needs for those with low (*β* = 0.26, *t*(430) = 2.90, *p* = 0.004, *sr*^2^ = 0.02 [0.00, 0.04]) and mean levels of fear of negative evaluation (*β* = 0.19, *t*(430) = 2.95, *p* = 0.003, *sr*^2^ = 0.02 [0.00, 0.04]) but not for those with high levels of fear of negative evaluation (*β* = 0.11, *t*(430) = 1.21, *p* = 0.228, *sr*^2^ = 0.00 [0.00, 0.01]). In other words, for participants at the mean and low levels of fear of negative evaluation, those in the Uberball condition reported significantly higher levels of fundamental need fulfillment than their counterparts in the Cyberball inclusion condition.

For those having received *positive* feedback, the condition term predicted fundamental needs for those at mean levels (*β* = 0.17, *t*(430) = 2.63, *p* = 0.009, *sr*^2^ = 0.01 [0.00, 0.03]) and high levels of fear of negative evaluation (*β* = 0.35, *t*(430) = 3.72, *p* < 0.001, *sr*^2^ = 0.03 [0.00, 0.06]) and not for those with low levels of fear of negative evaluation (*β* = −0.01, *t*(430) = −0.12, *p* = 0.905, *sr*^2^ = 0.00 [0.00, 0.00]). In other words, for participants at the mean and high levels of fear of negative evaluation, those in the Uberball condition reported significantly higher levels of fundamental need fulfillment than those in the Cyberball inclusion condition.

We also tested the “feedback × condition × fear of negative evaluation” interaction for each of the individual fundamental needs. The three-way interaction term significantly predicted *belongingness* (*β* = 0.44, *t*(430) = 2.38, *p* = 0.018, *sr*^2^ = 0.01 [0.00, 0.03]), *self-esteem* (*β* = 0.58, *t*(430) = 3.19, *p* = 0.002, *sr*^2^ = 0.02 [0.00, 0.04]), and *meaningful existence* (*β* = 0.60, *t*(430) = 3.25, *p* = 0.001, *sr*^2^ = 0.02 [0.00, 0.04]) but was not significant for *control* (*β* = 0.30, *t*(430) = 1.63, *p* = 0.103, *sr*^2^ = 0.01 [0.00, 0.02]).

## Discussion

The central objective of this research was to further understand the effects of the Uberball condition on fulfilling fundamental needs after providing either ambiguous or positive feedback to participants. Two main conclusions stem from the current study. First, preferential inclusion (Uberball condition) increases fundamental need fulfillment and relational value significantly more than ordinary inclusion (Cyberball inclusion condition). Second, whereas socially anxious individuals (with a high fear of negative evaluation) generally report a lower satisfaction of fundamental needs, the combination of receiving positive social feedback followed by experiencing ordinary inclusion (Cyberball inclusion condition) greatly exacerbates this tendency.

The first conclusion stems from the results showing that overall, participants who were the target of preferential inclusion (Uberball condition) reported higher levels of fundamental need fulfillment and perceived relational value than those who took part in an ordinary “social participation” task (Cyberball inclusion condition). This result conceptually replicates and extends Simard and Dandeneau (2018) by showing the additional effects on perceived relational value—an important mediating element of one’s feelings of personal self-esteem and self-worth (Leary, 2005).

Our second conclusion stems from the results of a three-way interaction between feedback condition, inclusion condition, and level of social insecurity showing that the expected negative relationship between fear of negative evaluation and fundamental need fulfillment is considerably stronger after receiving positive social feedback followed by experiencing ordinary inclusion (Cyberball inclusion condition). Interestingly, the effect was significant for the same three individual needs as in previous research: belongingness, self-esteem, and meaningful existence, and it was not significant for the control subscale (Simard and Dandeneau, 2018). Next, we explore this three-way interaction in more detail.

### Unmet expectations

Figure 2 suggests that in the ambiguous feedback condition, Uberball relates to higher fundamental need fulfillment relative to Cyberball inclusion for people with low or average levels of social insecurity (as confirmed by the simple slopes). In the positive feedback condition, both inclusion conditions relate to high fundamental need fulfillment in socially secure individuals, but for socially insecure individuals, Cyberball inclusion leads to lower levels of fundamental need fulfillment than the Uberball condition.

Accordingly, it seems that for those in the Cyberball inclusion condition, one’s level of social insecurity influences one’s emotional responses to receiving positive or ambiguous social feedback. Specifically, relative to ambiguous feedback, positive feedback appears to reduce fundamental need fulfillment in people with high social insecurity and to increase it in people with low social insecurity. We also know from our results that positive feedback led to higher anticipation of social acceptance relative to ambiguous feedback (a medium effect-sized difference). Thus, one interpretation of these results is that for socially insecure people, positive feedback may raise their social expectations of future social situations but that these expectations lead to disappointment when they are “merely included” in the group as opposed to being a highly valued member as in the Uberball condition.^4^ Although many would have their expectations unmet, socially anxious individuals may be particularly sensitive to it, highlighting the importance of expectation violations for this group of people (Wesselmann et al., 2017).

According to the temporal need-threat model of ostracism, detecting ostracism requires only the slightest representation of ostracism, and over-detection of ostracism likely serves an evolutionary purpose (Williams, 2009). However, some are more sensitive, hypervigilant, and overactive to social ostracism. Social exclusion makes people interpret neutral information as hostile (DeWall et al., 2009), and this tendency may be accentuated in socially insecure people. Although everyone tends to react negatively to negative feedback, socially hypersensitive people, for example, also tend to respond negatively to ambiguous feedback or even simply to the *absence* of positive feedback (Cikara and Girgus, 2010; Yang and Girgus, 2018). Because socially insecure individuals acutely fear negative social appraisals, they may interpret ordinary social inclusion negatively to confirm their chronic fears, à la self-fulfilling prophecy (Stinson et al., 2009, 2011). For example, for people with borderline personality disorder, being socially included through the Cyberball inclusion condition is not enough as they still feel rejected unless they experience extreme inclusion through a variant of the Uberball condition termed overinclusion (De Panfilis et al., 2015). Whereas healthy controls experienced as much rejection-related emotions, anxiety, and sadness during social participation (Cyberball inclusion) than during overinclusion, people with borderline personality disorder experienced substantially more rejection-related emotions, anxiety, and sadness after “mere inclusion/social participation” than after overinclusion. Given that people with borderline personality disorder typically have a higher fear of negative evaluation (Weinbrecht et al., 2020), it is possible that there is a similar dynamic at play in the current results—the “mere inclusion/social participation” in Cyberball inclusion simply did not live up to the social expectations created by positive feedback manipulation.

Socially secure people, on the other hand, may benefit from positive feedback relative to ambiguous feedback even when they are not preferentially included perhaps because they are better able to separate the social feedback component from the group’s behavior. These individuals probably have their fundamental needs already fulfilled and therefore are not actively trying to restore their needs, making them content even when their social value is not heightened (Hales and Williams, 2021).

Finally, the Uberball condition, interestingly, seems to eradicate the expectations contingencies. Whether participants receive positive or ambiguous social feedback seems to make little difference on the slope of fear of negative evaluation. Consistent with findings with borderline personality disorder and overinclusion (De Panfilis et al., 2015; Hales and Williams, 2021), the Uberball condition’s effect may come from the preferential inclusion they experience matching their positive expectations following positive feedback or eliminating doubt of one’s social value after ambiguous feedback.

### Limitations

This study carries a few limitations. First, there are known limitations to online samples from MTurk, CloudResearch, and the like (e.g., Aruguete et al., 2019). Second, the social feedback consisted of written conversation scripts, which may lack ecological validity and the “realness” of social interactions. Future research would benefit from replicating the current findings using more ecologically valid social interactions (e.g., confederates). Third, because we did not have a “no feedback” group (i.e., a group that did not receive any feedback) and a “no social interaction/inclusion” group (i.e., a group that completed a neutral task alone, as in Simard and Dandeneau, 2018), it is difficult to say whether participating in any social participation task (i.e., Cyberball inclusion or Uberball conditions) is better than *not* participating in a social participation task at all (i.e., completing a task alone). Thus, although the current data allow us to suggest general conclusions, we can only speculate as to the nature of the specific dynamics at play.

## Conclusion

This study adds to the evidence suggesting that social participation and preferential social inclusion constitute separate processes that lead to distinct psychological outcomes (e.g., fundamental needs). It also suggests that the social context under which social inclusion is experienced may influence one’s emotional response to this social inclusion, especially for socially insecure individuals. In particular, socially insecure individuals may be motivated to restore fundamental needs by building positive expectations following initial positive feedback but end up even more disappointed when reality does not live up to their expectations. Ultimately, the Uberball condition constitutes a timely addition to the social scientist’s toolbox for further exploring the dynamics of social inclusion.

## Data availability statement

The data, analysis scripts, and supplemental materials for this study are available on the Open Science Framework at: https://osf.io/cmg3z/.

## Ethics statement

The studies involving humans were approved by the Comité institutionnel d’éthique de la recherche avec des êtres humains (CIEREH) at Université du Québec à Montréal (UQAM). The studies were conducted in accordance with the local legislation and institutional requirements. Participants first read the description of the study before providing their informed consent to participate.

## Author contributions

RT: Data curation, Formal analysis, Investigation, Software, Visualization, Writing – original draft, Writing – review & editing. FD-C: Conceptualization, Data curation, Investigation, Methodology, Project administration, Writing – original draft. SD: Conceptualization, Funding acquisition, Investigation, Methodology, Project administration, Resources, Supervision, Writing – original draft, Writing – review & editing.

## Footnotes

^1^Available on the Open Science Framework at https://osf.io/cmg3z/

^2^Square brackets represent 95% confidence intervals throughout this manuscript.

^3^We report the semi-partial correlation squared (*sr^2^*) and its 95% confidence interval as an index of the effect size. The *sr^2^* allows us to quantify the unique contribution (proportion of variance explained) of an independent variable on the dependent variable, over and above the other variables in the model. The s*r^2^* is often considered a better indicator of the practical relevance of a variable.

^4^A mediation analysis revealed that anticipation of social acceptance partly mediates (28%) the relationship between fear of negative evaluation and fundamental needs, *b* (for the indirect effect) = −0.09 [−0.13, −0.04], *p* < 0.001. Another mediation analysis suggests that anticipation of social acceptance also partly mediates the relationship between *feedback* and fundamental needs, *b* (for the indirect effect) = 0.21 [0.12, 0.31], *p* < 0.001. Essentially, while positive feedback leads to higher anticipation of social acceptance, anticipation leads to higher fundamental needs (however, a negative direct effect prevents the total effect from reaching significance).

## References

[ref1] Ahlmann-Eltze C. (2019). ggsignif: significance brackets for “ggplot2” (R package version 0.6.0). Available at: https://CRAN.R-project.org/package=ggsignif.

[ref2] Aiken L. S. West S. G. (1991). Multiple regression: testing and interpreting interactions. Newbury Park, CA: Sage Publications, Inc.

[ref3] Amir N. Beard C. Bower E. (2005). Interpretation bias and social anxiety. Cogn. Ther. Res. 29, 433–443. doi: 10.1007/s10608-005-2834-5

[ref4] Anthony D. B. Wood J. V. Holmes J. G. (2007). Testing sociometer theory: self-esteem and the importance of acceptance for social decision-making. J. Exp. Soc. Psychol. 43, 425–432. doi: 10.1016/j.jesp.2006.03.002

[ref5] Aruguete M. S. Huynh H. Browne B. L. Jurs B. Flint E. McCutcheon L. E. (2019). How serious is the “carelessness” problem on mechanical turk? Int. J. Soc. Res. Methodol. 22, 441–449. doi: 10.1080/13645579.2018.1563966

[ref6] Baumeister R. F. Bratslavsky E. Finkenauer C. Vohs K. D. (2001). Bad is stronger than good. Rev. Gen. Psychol. 5, 323–370. doi: 10.1037/1089-2680.5.4.323

[ref7] Beard C. Amir N. (2008). A multi-session interpretation modification program: changes in interpretation and social anxiety symptoms. Behav. Res. Ther. 46, 1135–1141. doi: 10.1016/j.brat.2008.05.012, PMID: 18675400 PMC3569034

[ref8] Ben-Shachar M. S. Lüdecke D. Makowski D. (2020). effectsize: estimation of effect size indices and standardized parameters. J. Open Source Softw. 5:2815. doi: 10.21105/joss.02815

[ref9001] Ben-Shachar M. S. Makowski D. Lüdecke D. Patil I. Wiernik B. M. Thériault R. (2022). effectsize: Indices of effect size. (R package version 0.8.2) [Computer software]. https://easystats.github.io/effectsize

[ref9] Cameron J. J. Stinson D. A. Gaetz R. Balchen S. (2010). Acceptance is in the eye of the beholder: self-esteem and motivated perceptions of acceptance from the opposite sex. J. Pers. Soc. Psychol. 99, 513–529. doi: 10.1037/a0018558, PMID: 20649372

[ref10] Carleton R. N. Collimore K. C. Asmundson G. J. G. (2007). Social anxiety and fear of negative evaluation: construct validity of the BFNE-II. J. Anxiety Disord. 21, 131–141. doi: 10.1016/j.janxdis.2006.03.010, PMID: 16675196

[ref11] Carleton R. N. McCreary D. R. Norton P. J. Asmundson G. J. (2006). Brief fear of negative evaluation scale-revised. Depress. Anxiety 23, 297–303. doi: 10.1002/da.20142, PMID: 16688736

[ref12] Champely S. (2020). pwr: basic functions for power analysis (R package version 1.3-0). Available at: https://CRAN.R-project.org/package=pwr

[ref13] Chen J. Short M. Kemps E. (2020). Interpretation bias in social anxiety: a systematic review and meta-analysis. J. Affect. Disord. 276, 1119–1130. doi: 10.1016/j.jad.2020.07.121 32777650

[ref14] Cikara M. Girgus J. S. (2010). Unpacking social hypersensitivity: vulnerability to the absence of positive feedback. Pers. Soc. Psychol. Bull. 36, 1409–1423. doi: 10.1177/0146167210383288, PMID: 20841434

[ref15] Curtin J. (2018). lmSupport: support for linear models (R package version 2.9.13). Available at: https://CRAN.R-project.org/package=lmSupport.

[ref16] De Panfilis C. Riva P. Preti E. Cabrino C. Marchesi C. (2015). When social inclusion is not enough: implicit expectations of extreme inclusion in borderline personality disorder. Personal. Disord. Theory Res. Treat. 6, 301–309. doi: 10.1037/per0000132 26147068

[ref17] Delacre M. Lakens D. Leys C. (2017). Why psychologists should by default use Welch’s *t*-test instead of student’s *t*-test. Int. Rev. Soc. Psychol. 30, 92–101. doi: 10.5334/irsp.82

[ref18] DeWall C. N. Twenge J. M. Gitter S. A. Baumeister R. F. (2009). It's the thought that counts: the role of hostile cognition in shaping aggressive responses to social exclusion. J. Pers. Soc. Psychol. 96, 45–59. doi: 10.1037/a0013196, PMID: 19210063 PMC2775524

[ref19] Downey G. Feldman S. I. (1996). Implications of rejection sensitivity for intimate relationships. J. Pers. Soc. Psychol. 70, 1327–1343. doi: 10.1037/0022-3514.70.6.1327, PMID: 8667172

[ref9002] Dvir M. Kelly J. R. Williams K. D. (2019). Is inclusion a valid control for ostracism? J. Soc. Psychol. 159, 106–111. doi: 10.1080/00224545.2018.1460301 29621427

[ref20] Eisenberger N. I. Lieberman M. D. Williams K. D. (2003). Does rejection hurt? An fMRI study of social exclusion. Science 302, 290–292. doi: 10.1126/science.1089134, PMID: 14551436

[ref21] Hales A. H. Williams K. D. (2021). “Social ostracism: theoretical foundations and basic principles” in Social psychology: handbook of basic principles. eds. Van Lange P. A. M. Tory Higgins E. Kruglanski A. W. 3rd ed (New York, NY, US: The Guilford Press), 337–349.

[ref22] Hayes A. F. (2018). Introduction to mediation, moderation, and conditional process analysis: a regression-based approach Guilford Press.

[ref23] Heinrichs N. Hofmann S. G. (2001). Information processing in social phobia: a critical review. Clin. Psychol. Rev. 21, 751–770. doi: 10.1016/S0272-7358(00)00067-2, PMID: 11434229

[ref24] Hirsch C. R. Clark D. M. Mathews A. (2006). Imagery and interpretations in social phobia: support for the combined cognitive biases hypothesis. Behav. Ther. 37, 223–236. doi: 10.1016/j.beth.2006.02.001, PMID: 16942974

[ref25] Jamieson J. P. Harkins S. G. Williams K. D. (2010). Need threat can motivate performance after ostracism. Personal. Soc. Psychol. Bull. 36, 690–702. doi: 10.1177/0146167209358882, PMID: 20388870

[ref26] Kassambara A. (2019). ggpubr: “ggplot2” based publication ready plots (R package version 0.2.2). Available at: https://CRAN.R-project.org/package=ggpubr.

[ref27] Kercher K. (1992). Assessing subjective well-being in the old-old: the PANAS as a measure of orthogonal dimensions of positive and negative affect. Res. Aging 14, 131–168. doi: 10.1177/0164027592142001

[ref28] Kirby K. N. Gerlanc D. (2013). bootES: an R package for bootstrap confidence intervals on effect sizes. Behav. Res. Methods 45, 905–927. doi: 10.3758/s13428-013-0330-5, PMID: 23519455

[ref29] Leary M. R. (2005). Sociometer theory and the pursuit of relational value: getting to the root of self-esteem. Eur. Rev. Soc. Psychol. 16, 75–111. doi: 10.1080/10463280540000007

[ref30] Leary M. R. (2010). “Affiliation, acceptance, and belonging: the pursuit of interpersonal connection” in Handbook of social psychology, vol. 2. eds. Van Lange P. A. M. Tory Higgins E. Kruglanski A. W. 5th ed (Hoboken, NJ, US: John Wiley & Sons, Inc), 864–897.

[ref31] Leary M. R. Baumeister R. F. (2000). “The nature and function of self-esteem: sociometer theory” in Advances in experimental social psychology, vol. 32 (San Diego, CA, US: Academic Press), 1–62.

[ref32] Leys C. Ley C. Klein O. Bernard P. Licata L. (2013). Detecting outliers: do not use standard deviation around the mean, use absolute deviation around the median. J. Exp. Soc. Psychol. 49, 764–766. doi: 10.1016/j.jesp.2013.03.013

[ref33] Litman L. Robinson J. Abberbock T. (2017). TurkPrime.com: a versatile crowdsourcing data acquisition platform for the behavioral sciences. Behav. Res. Methods 49, 433–442. doi: 10.3758/s13428-016-0727-z, PMID: 27071389 PMC5405057

[ref34] Long J. A. (2019). interactions: comprehensive, user-friendly toolkit for probing interactions (R package version 1.1.0). Available at: https://CRAN.R-project.org/package=interactions.

[ref35] Makowski D. Lüdecke D. Ben-Shachar M. S. Patil I. Wiernik Brenton M. Thériault R. (2022). report: from R to your manuscript. (R package version 0.5.5.3). Available at: https://easystats.github.io/report.

[ref36] Mangiafico S. (2020). rcompanion: functions to support extension education program evaluation (R package version 2.3.26). Available at: https://CRAN.R-project.org/package=rcompanion.

[ref37] Millisecond Software LLC. (2016). *Inquisit* [computer software]. Available at: https://www.millisecond.com/.

[ref38] Parent M. C. (2013). Handling item-level missing data: simpler is just as good. Couns. Psychol. 41, 568–600. doi: 10.1177/0011000012445176

[ref39] Peterson R. A. (2021). Finding optimal normalizing transformations via bestNormalize. R J. 13, 310–329. doi: 10.32614/RJ-2021-041

[ref40] Peterson R. A. Cavanaugh J. E. (2020). Ordered quantile normalization: a semiparametric transformation built for the cross-validation era. J. Appl. Stat. 47, 2312–2327. doi: 10.1080/02664763.2019.1630372, PMID: 35707424 PMC9042069

[ref41] R Core Team. (2022). R: a language and environment for statistical computing (version 4.2.1) [Computer software]. R Foundation for Statistical Computing, Vienna, Austria. Available at: https://www.R-project.org/.

[ref42] Revelle W. (2018). psych: procedures for personality and psychological research (R package version 2.1.6). Northwestern University, Evanston, Illinois, USA, Available at: https://CRAN.R-project.org/package=psych.

[ref43] Rosenberg M. (1965). Society and the adolescent self-image. Princeton, NJ, US: Princeton University Press. Available at: http://www.jstor.org/stable/j.ctt183pjjh.

[ref44] Schröder-Abé M. Rudolph A. Wiesner A. Schütz A. (2007). Self-esteem discrepencies and defensive reactions to social feedback. Int. J. Psychol. 42, 174–183. doi: 10.1080/00207590601068134

[ref45] Simard V. Dandeneau S. (2018). Revisiting the Cyberball inclusion condition: fortifying fundamental needs by making participants the target of specific inclusion. J. Exp. Soc. Psychol. 74, 38–42. doi: 10.1016/j.jesp.2017.08.002

[ref46] Slowikowski K. Schep A. Hughes S. Lukauskas S. Irisson J.-O. Kamvar Z. N.. (2018). ggrepel: automatically position non-overlapping text labels with “ggplot2” (R package version 0.9.1). Available at: https://CRAN.R-project.org/package=ggrepel.

[ref47] Stekhoven D. J. (2022). missForest: nonparametric missing value imputation using random forest (R package version 1.5). Available at: https://cran.r-project.org/package=missForest.

[ref48] Stekhoven D. J. Bühlmann P. (2012). MissForest—non-parametric missing value imputation for mixed-type data. Bioinformatics 28, 112–118. doi: 10.1093/bioinformatics/btr597, PMID: 22039212

[ref49] Stinson D. A. Cameron J. J. Wood J. V. Gaucher D. Holmes J. G. (2009). Deconstructing the “reign of error”: interpersonal warmth explains the self-fulfilling prophecy of anticipated acceptance. Personal. Soc. Psychol. Bull. 35, 1165–1178. doi: 10.1177/0146167209338629, PMID: 19571273

[ref50] Stinson D. A. Logel C. Shepherd S. Zanna M. P. (2011). Rewriting the self-fulfilling prophecy of social rejection: self-affirmation improves relational security and social behavior up to 2 months later. Psychol. Sci. 22, 1145–1149. doi: 10.1177/0956797611417725, PMID: 21813799

[ref51] Thériault R. (2023). rempsyc: convenience functions for psychology. J. Open Source Softw. 8:5466. doi: 10.21105/joss.05466

[ref52] Thériault R. Ben-Shachar M. S. Patil I. Lüdecke D. Wiernik B. M. Makowski D. (2023a). Check your outliers! An introduction to identifying statistical outliers in R with easystats. PsyArXiv, 1–9. doi: 10.31234/osf.io/bu6nt 38528245

[ref53] Thériault R. Dion-Cliche F. Dandeneau S. (2023b). Unmet expectations: social inclusion and the interaction between social anxiety and ambiguous or positive feedback. PsyArXiv, 1–36. doi: 10.31234/osf.io/xc4g7 PMC10728674 38115983

[ref54] Tierney N. (2017). visdat: visualising whole data frames. J. Open Source Softw. 2:355. doi: 10.21105/joss.00355

[ref55] Tierney N. Cook D. McBain M. Fay C. (2021). naniar: data structures, summaries, and visualisations for missing data (R package version 0.6.1). Available at: https://CRAN.R-project.org/package=naniar.

[ref56] Tversky A. Kahneman D. (1974). Judgment under uncertainty: heuristics and biases. Science 185, 1124–1131. doi: 10.1126/science.185.4157.1124 17835457

[ref57] van Ginkel J. R. Linting M. Rippe R. C. A. van der Voort A. (2020). Rebutting existing misconceptions about multiple imputation as a method for handling missing data. J. Pers. Assess. 102, 297–308. doi: 10.1080/00223891.2018.1530680, PMID: 30657714

[ref58] Weinbrecht A. Roepke S. Renneberg B. (2020). Fear of positive evaluation in borderline personality disorder. PLoS One 15:e0237944. doi: 10.1371/journal.pone.0237944, PMID: 32817666 PMC7444514

[ref59] Wesselmann E. Wirth J. Bernstein M. (2017). Expectations of social inclusion and exclusion. Front. Psychol. 8:112. doi: 10.3389/fpsyg.2017.00112, PMID: 28220092 PMC5292417

[ref60] Wickham H. (2016). ggplot2: elegant graphics for data analysis (R package version 3.3.3). Springer-Verlag: New York. Available at: https://ggplot2.tidyverse.org.

[ref61] Wickham H. François R. Henry L. Müller K. (2021). dplyr: a grammar of data manipulation (R package version 1.0.5). Available at: https://CRAN.R-project.org/package=dplyr.

[ref62] Williams K. D. (2007). Ostracism. Annu. Rev. Psychol. 58, 425–452. doi: 10.1146/annurev.psych.58.110405.085641 16968209

[ref63] Williams K. D. (2009). “Chapter 6 ostracism: a temporal need-threat model” in Advances in experimental social psychology, vol. 41 (San Diego, CA, US: Academic Press), 275–314.

[ref64] Williams K. D. Cheung C. K. Choi W. (2000). Cyberostracism: effects of being ignored over the internet. J. Pers. Soc. Psychol. 79, 748–762. doi: 10.1037/0022-3514.79.5.748, PMID: 11079239

[ref65] Williams K. D. Jarvis B. (2006). Cyberball: a program for use in research on interpersonal ostracism and acceptance. Behav. Res. Methods 38, 174–180. doi: 10.3758/BF03192765, PMID: 16817529

[ref66] Williams K. D. Nida S. A. (2011). Ostracism: consequences and coping. Curr. Dir. Psychol. Sci. 20, 71–75. doi: 10.1177/0963721411402480

[ref67] Yang K. Girgus J. S. (2018). Individual differences in social hypersensitivity predict the interpretation of ambiguous feedback and self-esteem. Personal. Individ. Differ. 135, 316–327. doi: 10.1016/j.paid.2018.07.022

[ref68] Yun H. J. Hyun M.-H. (2023). Effects of social anxiety level on negative interpretation bias in ambiguous social situations: focused on relational intimacy. Stress 31, 11–17. doi: 10.17547/kjsr.2023.31.1.11

